# Onset age is a risk factor for refractory pediatric IgA vasculitis: a retrospective cohort study

**DOI:** 10.1186/s12969-020-00480-3

**Published:** 2020-11-10

**Authors:** Chun-Hua Liao, Melody Tsai, Yao-Hsu Yang, Bor-Luen Chiang, Li-Chieh Wang

**Affiliations:** 1 Department of Pediatrics, National Taiwan University Hospital, No.8, Chung Shan South Road, Taipei, 10002 Taiwan; 2 Department of Medical Research, National Taiwan University Hospital, Taipei, Taiwan

**Keywords:** Pediatric IgA vasculitis, Onset age, Corticosteroid dependence, Refractory, Renal involvement

## Abstract

**Background:**

Though outcome differences between children and adults with immunoglobulin A vasculitis (IgAV) has been well documented, it remains unclear if disease features in pediatric IgAV patients vary with onset age. We aimed to explore clinical features and prognosis of pediatric IgAV stratified by onset age.

**Methods:**

We retrospectively reviewed records of patients under 18 years old diagnosed with IgAV from January 1999 to December 2018 in one tertiary medical center in Taiwan. Patients were grouped by onset age: ≤ 6 years old, 6–12 years old (> 6, ≤ 12), and 12–18 years old (> 12, < 18). Demographics, laboratory data, incidence of gastrointestinal, renal, and joint involvement, corticosteroid dependence, recurrence, and refractory disease were analyzed. Recurrence was defined as disease flare-up after complete remission and discontinuation of all medications for at least 3 months. Corticosteroid dependence was defined by more than 6 weeks of daily oral corticosteroid intake. Refractory disease was defined as not achieving complete remission 6 months after disease onset. Statistical analysis was performed using R software (v3.6.0).

**Results:**

There were 484 IgAV patients, with an onset age of 6.10 (4.72–8.58) (median (IQR)) years old. There were 234 (48.3%) patients ≤6 years old, 210 (43.4%) 6–12 years old, and 40 (8.3%) 12–18 years old. One hundred and thirty (26.9%) patients had renal involvement, which was more frequent in older children (≤ 6 years old, 18.4%; 6–12 years old, 31.0%; 12–18 years old, 55.0%; *p* <  0.001). There were 361 patients (74.6%) with joint involvement; younger children were affected more frequently (≤ 6 years old, 82.1%; 6–12 years old, 71.9%; 12–18 years old, 45.0%; *p* <  0.001). Gastrointestinal involvement was present in 311 (64.3%) patients, showing no difference among age groups. There were 46 patients (9.5%) with recurrent IgA vasculitis, 136 (28.1%) with corticosteroid dependent and 76 (15.7%) with refractory disease. Corticosteroid dependence and refractory disease occurred more frequently as onset age increased (*p* <  0.001).

**Conclusion:**

Pediatric IgAV with different onset ages are associated with distinct clinical manifestations and outcomes. The risk of developing corticosteroid dependence, refractory disease and renal involvement increased with onset age.

**Supplementary Information:**

The online version contains supplementary material available at 10.1186/s12969-020-00480-3.

## Background

Immunoglobulin A vasculitis (IgAV), also known as Henoch–Schönlein purpura, is the most common vasculitis in children, with an annual incidence of 13–20 per 100,000 children, and a peak age of onset between 4 to 6 years old [1–3]. The male to female ratio is around 1.1 [2, 3]. Though the exact pathogenic mechanisms remain unclear, previous studies suggested that IgAV might be associated with infection, supported by observations of seasonality and preceding upper airway infections [4].

The pathological hallmarks in IgAV are deposition of immunoglobulin A (IgA) at the walls of small vessels and perivascular neutrophil infiltration. In previous in vitro studies, IgA anti-endothelial cell antibodies from IgAV patients activated endothelial cells to produce cytokines such as interleukin-8 (IL-8), and further resulted in inflammatory responses and neutrophil migration [5]. These findings suggest that the interaction between IgA, neutrophils, and endothelial cells might play a major role in the pathogenesis of IgAV [6].

Typical symptoms and signs of IgAV include non-blanchable purpura (mostly over the lower extremities), arthralgia/arthritis, abdominal cramping pain, and hematuria/proteinuria. Though its prognosis is considered benign in children, adults with IgAV have a higher risk of developing refractory renal, gastrointestinal (GI), and neurological disease. Compared to children, adults with IgAV have distinct epidemiology and disease manifestations. The annual incidence is lower, around 0.34 to 1.4 per 100,000 persons [7], and the male to female ratio is higher, up to 1.5 [8]. There is a higher frequency of renal involvement, around 50–85% [9–12], a higher risk of progression to renal insufficiency, and increased mortality of all causes [13].

While outcome differences between children and adults with IgAV have been well documented, it is less clear whether and how disease features in pediatric IgAV patients vary with onset age. We aimed to clarify the differences between children of different age groups in terms of disease presentation and prognosis.

## Methods

We retrospectively reviewed the medical records of the patients under 18 years old with a diagnosis of IgAV from January 1999 to December 2018 at one tertiary medical center in Taiwan. The diagnosis of IgAV was based on validated criteria defined by the 2010 European League Against Rheumatism/Pediatric Rheumatology International Trials Organization/Pediatric Rheumatology European Society (EULAR/PRINTO/PRES) [14]. Patients were divided into three groups according to onset age: ≤ 6 years old, 6–12 years old (> 6, ≤ 12), and 12–18 years old (> 12, < 18). We retrieved epidemiologic characteristics, laboratory data at onset, organ involvement, prescribed medications, including: corticosteroids (CS), non-steroidal anti-inflammatory drugs (NSAIDs), and disease-modifying anti-rheumatic drugs (DMARDs), total CS exposure duration, total CS cumulative dose, exposure duration of high dose CS (equivalent dose of oral prednisolone ≥1 mg/kg/day), recurrence rate, CS dependent rate, and refractory rate.

### Definition of renal involvement

Renal involvement was defined as hematuria and/or proteinuria. Hematuria was defined by the presence of at least five red blood cells (RBCs) per high power field or RBC ≥ 2+ on dipstick or RBC casts in the urinary sediment. Proteinuria was defined as protein loss of more than 0.3 g per 24 h or > 30 mmol/mg of urine albumin to creatinine ratio on a spot morning sample.

### Definition of recurrent IgAV, refractory IgAV, and CS dependence

Recurrent IgAV was defined as disease flare-up after complete remission and discontinuation of all medications for at least 3 months. CS dependence was defined by more than 6 weeks of daily oral CS intake. Refractory disease was defined as not achieving complete remission 6 months after disease onset. Complete remission was defined as the resolution of skin purpura, arthralgia/arthritis, and abdominal pain, combined with normal renal function and absence of proteinuria and hematuria, as well as discontinuation of all medication.

### Statistical analysis

Laboratory data were presented as median (interquartile range, IQR). Continuous data were compared using the Kruskal-Wallis test. We compared categorical variables and proportions by using the chi-square test. Univariate and multivariate regression models were used to identify significant factors associated with clinical outcomes. To model the relationship between outcome probabilities and onset age, allowing for nonlinear effects, we used logistic regression with restricted cubic spline basis functions, with four evenly spaced knots [15]. We also tested modeling with three to six knots, which yielded highly similar results. The optimal threshold in receiver operating characteristic (ROC) curve analysis was chosen by maximizing Youden’s index. A threshold of *P* <  0.05 was used for statistical significance. Statistical analyses were conducted with R software (version 3.6.0), using the rms, pROC, and cutpointr packages. The ggplot2 package was used for visualizations.

## Results

### Patient characteristics

There were 484 patients enrolled in this retrospective study. Demographic data and clinical characteristics of all patients and the three subgroups are summarized in Table 1. There were 252 boys among all patients, and the male to female ratio was 1.09. The onset age was 6.10 (IQR:4.72–8.58) years old. Among all patients, 234 (48.3%) patients were ≤ 6 years old; 210 (43.4%) of them were 6–12 years old, and 40 (8.3%) were 12–18 years old. The percentage of male patients showed no significant difference among the three subgroups. Overall, preceding infection symptoms and/or signs were noted in 304 (62.8%) patients, but they occurred more frequently in younger age groups (≤ 6 years old, 71.8%; 6–12 years old, 56.2%; 12–18 years old, 40.0%; *P* <  0.001). More than half of the cases occurred in autumn and winter, though without statistical significance. (*P* = 0.670, data not shown, see Additional file 1: Table S1).

**Table 1:** Demographic and clinical manifestations in pediatric IgA vasculitis according to different onset ages

	Total (*n* = 484)	≤6 Y (*n* = 234)	6–12 Y (*n* = 210)	12–18 Y^a^ (*n* = 40)	*P* value
Onset age, years old	6.10 (4.72–8.58)	4.68 (3.83–5.27)	7.91 (6.88–9.55)	14.48 (12.97–15.90)	
Male sex	252 (52.1%)	122 (52.1%)	110 (52.4%)	20 (50.0%)	0.962
Preceding infection	304 (62.8%)	168 (71.8%)	118 (56.2%)	16 (40.0%)	**< 0.001**
Manifestation					
Skin purpura	484 (100%)	234 (100%)	210 (100%)	40 (100%)	NA
Arthralgia/arthritis	361 (74.6%)	192 (82.1%)	151 (71.9%)	18 (45.0%)	**< 0.001**
CNS/PNS involvement	5 (1.0%)	2 (0.9%)	2 (1.0%)	1 (2.5%)	NA
GI involvement	311 (64.3%)	156 (66.7%)	130 (61.9%)	25 (62.5%)	0.562
Abdominal pain	294 (60.7%)	147 (62.8%)	123 (58.6%)	24 (60.0%)	0.654
Vomiting	100 (20.7%)	50 (21.4%)	44 (21.0%)	6 (15.0%)	0.649
Diarrhea	34 (7.0%)	11 (4.7%)	18 (8.6%)	5 (12.5%)	0.103
GI bleeding	42 (8.7%)	23 (9.8%)	15 (7.1%)	4 (10.0%)	0.576
Positive stool occult blood	111 (22.9%)	56 (23.9%)	44 (21.0%)	11 (27.5%)	0.586
Renal involvement	130 (26.9%)	43 (18.4%)	65 (31.0%)	22 (55.0%)	**< 0.001**
Microscopic hematuria	129 (26.7%)	43 (18.5%)	64 (30.5%)	22 (55.0%)	**< 0.001**
Gross hematuria	10 (20.7%)	4 (1.7%)	6 (2.9%)	0	NA
Non-nephrotic proteinuria	24 (5.0%)	8 (3.4%)	13 (6.2%)	3 (7.5%)	0.301
Nephrotic syndrome	8 (1.7%)	2 (0.9%)	5 (2.4%)	1 (2.5%)	NA

Data shown are median (IQR) or number (%) of patients as appropriate

*Abbreviations*: *IgA* Immunoglobulin A, *CNS* Central nervous system, *PNS* Peripheral nervous system, *GI* Gastrointestinal, *NA* Not available

^a^ Patients were grouped by onset age: ≤ 6 Y (years old), 6–12 Y (> 6, ≤ 12 years old), and 12–18 Y (> 12, < 18 years old)

Renal involvement was noted in 130 (26.9%) patients, and it occurred more frequently in older children (≤ 6 years old, 18.4%; 6–12 years old, 31.0%; 12–18 years old, 55.0%; *P* <  0.001). Three hundred sixty-one patients (74.6%) had arthritis or arthralgia, and it was found more frequently in younger children (≤ 6 years old, 82.1%; 6–12 years old, 71.9%; 12–18 years old, 45.0%; P <  0.001). GI involvement was noted in 311 (64.3%) of the patients. There was no difference in the incidence of GI associated symptoms among the subgroups.

### Laboratory analysis

Hemoglobin (Hb), platelet (Plt), white blood cell count (WBC), neutrophil count, lymphocyte count, neutrophil to lymphocyte ratio (NLR), platelet to lymphocyte ratio (PLR), and serum IgA were compared among the three subgroups via Kruskal-Wallis test; the results are summarized in Table 2. Hb, lymphocyte count, NLR, PLR, and IgA showed significant differences among the three subgroups. Because normal values of hemoglobin, WBC, neutrophil count, lymphocyte count and level of IgA varied across different ages, we used age-corrected Z-scores to offset the possible age effect [16, 17]. After adjustment by age, the Z-score for lymphocyte count showed no significant difference over different age subgroups. As onset age increased, the Z-score of WBC increased, but the Z-scores of Hb and IgA declined significantly (Table 3). We did not adjust NLR and PLR by age, because there was no validated normal range for NLR and PLR of different ages.

**Table 2:** Laboratory parameters in pediatric IgA vasculitis patients in different onset age groups

	≤6 Y (*n* = 234)	6–12 Y (*n* = 210)	12–18 Y^a^ (*n* = 40)	*P* value
Hb (g/dl)	12.60 (11.9–13.3) (*n* = 178)	13.10 (12.30–13.70) (*n* = 161)	13.55 (12.95–14.40) (*n* = 24)	**< 0.001**
Plt (10^9^/L)	346.0 (284.0–427.0) (*n* = 178)	345.0 (300.0–406.0) (*n* = 161)	345.5 (260.8–389.8 (*n* = 24)	0.309
WBC(10^9^/L)	10,600 (8622–12,950) (*n* = 180)	10,615 (8478–13,365) (*n* = 158)	9900 (8358–11,545) (*n* = 24)	0.609
Neutrophil (10^9^/L)	6250 (4593–8177) (*n* = 177)	7166 (5115–10,156) (*n* = 151)	6171 (5229–8103) (*n* = 24)	**0.049**
Lymphocyte (10^9^/L)	3211.5 (2460–4147.1) (*n* = 177)	2598.7(1879.5–3348.9) (*n* = 150)	2195 (1645–2872) (*n* = 24)	**< 0.001**
NLR	1.91 (1.26–2.82) (*n* = 177)	2.95 (1.60–4.61) (*n* = 150)	2.68 (1.90–4.68) (*n* = 24)	**< 0.001**
PLR	108.59 (80.38–141.61) (*n* = 175)	126.89 (100.71–190.99) (*n* = 150)	154.00 (106.67–183.42) (*n* = 24)	**< 0.001**
IgA (mg/dL)	189.5 (137.8–259.0) (*n* = 168)	253.5 (217.0–340.5) (*n* = 154)	308.5 (231.0–395.8) (*n* = 30)	**< 0.001**
ANA≧1:80 positive	12/154	14/143	2/30	0.767
CRP≧1 mg/dL	63/151	42/120	12/19	0.059

Data are presented as median (IQR)

*Abbreviations*: *Hb* Hemoglobin, *Plt* Platelet, *WBC* White blood cells, *PLR* Platelet-to-lymphocyte ratio, *NLR* Neutrophil-to-lymphocyte ratio, *IgA* Immunoglobulin A, *ANA* Antinuclear antibody, *CRP* C-reactive protein

^a^ Patients were grouped by onset age: ≤ 6 Y (years old), 6–12 Y (> 6, ≤ 12 years old), and 12–18 Y (> 12, < 18 years old)

**Table 3:** Adjusted laboratory parameters in pediatric IgA vasculitis patients in different onset age groups

	≤6 Y (*n* = 234)	6–12 Y (*n* = 210)	12–18 Y^a^ (*n* = 40)	*P* value
Hb Z score	0.12 ± 2.11 (*n* = 178)	− 0.45 ± 1.07 (*n* = 161)	− 0.75 ± 1.70(*n* = 24)	**0.002**
WBC Z score	1.77 ± 1.53 (*n* = 180)	2.53 ± 2.32(*n* = 158)	3.15 ± 2.51 (*n* = 24)	**< 0.001**
Neutrophil Z score	1.76 ± 1.85 (*n* = 177)	2.30 ± 2.80(*n* = 151)	1.88 ± 2.16 (*n* = 24)	0.501
Lymphocyte Z score	−0.17 ± 1.08 (*n* = 177)	0.07 ± 1.67 (*n* = 150)	0.16 ± 1.04 (*n* = 24)	0.359
IgA Z score	4.36 ± 3.74 (*n* = 168)	3.10 ± 1.96 (*n* = 154)	2.56 ± 1.70 (*n* = 30)	**0.004**

Data are presented as mean ± SD

*Abbreviations*: *Hb* Hemoglobin, *WBC* White blood cells, *IgA* Immunoglobulin A

^a^ Patients were grouped by onset age: ≤ 6 Y (years old), 6–12 Y (> 6, ≤ 12 years old), and 12–18 Y (> 12, < 18 years old)

### Medication

The most commonly used medications were NSAIDs and CS, which were prescribed to 90.1 and 83.9% of the patients, respectively. Azathioprine (46.3%) was the most commonly prescribed DMARD, followed by cyclosporine (13.2%) and hydroxychloroquine (12.6%).

Twenty patients received methylprednisolone mini-pulse or pulse therapy (15 mg/kg/day for 2 days, with a maximum of 500 mg/day; or 30 mg/kg/day for 3 days, with a maximum of 1000 mg/day). Three patients received intravenous immunoglobulin (1 g/kg/day for 2 days); two patients received cyclophosphamide pulse therapy for nephrotic syndrome; one patient received rituximab for refractory abdominal pain and arthralgia.

The administrative rate of NSAIDs and CS among the three subgroups showed no significant difference. However, use of DMARDs was more frequent in children over 6 years old (≤ 6 years old, 36.4%; 6–12 years old, 60.9%; 12–18 years old, 71.4%; *P* <  0.001) (Table 4).

**Table 4:** Medication use in pediatric IgA vasculitis patients of different onset age

	Total (*n* = 484)	≤6 Y (*n* = 234)	6–12 Y (*n* = 210)	12–18 Y^a^ (*n* = 40)	*P* value
CS	406 (83.9%)	193 (82.5%)	179 (85.2%)	34 (85.0%)	0.718
NSAIDs	436 (90.1%)	216 (92.3%)	188 (89.5%)	32 (80.0%)	0.518
DMARDs	253 (52.3%)	91 (38.9%)	133 (63.3%)	29 (72.5%)	**< 0.001**
Azathioprine	224 (46.3%)	83 (35.5%)	119 (56.7%)	22 (55.0%)	**< 0.001**
Cyclosporine	64 (13.2%)	20 (8.5%)	33 (15.7%)	11 (27.5%)	**0.002**
hydroxychloroquine	61 (12.6%)	22 (9.4%)	29 (13.8%)	10 (25.0%)	**0.018**
Others^b^	32 (6.6%)	3 (1.3%)	18 (8.6%)	11 (27.5%)	**< 0.001**

Data shown are number (%) of patients

*Abbreviations*: *IgA* Immunoglobulin A, *CS* Corticosteroid, *NSAIDs* Non-steroidal anti-inflammatory drugs, *DMARDs* Disease-modifying anti-rheumatic drugs

^a^ Patients were grouped by onset age: ≤ 6 Y (years old), 6–12 Y (> 6, ≤ 12 years old), and 12–18 Y (> 12, < 18 years old)

^b^ Other DMARDs included mesalazine, sulfasalazine, dapsone, colchicine, methotrexate, and mycophenolate mofetil

**Fig. 1:**
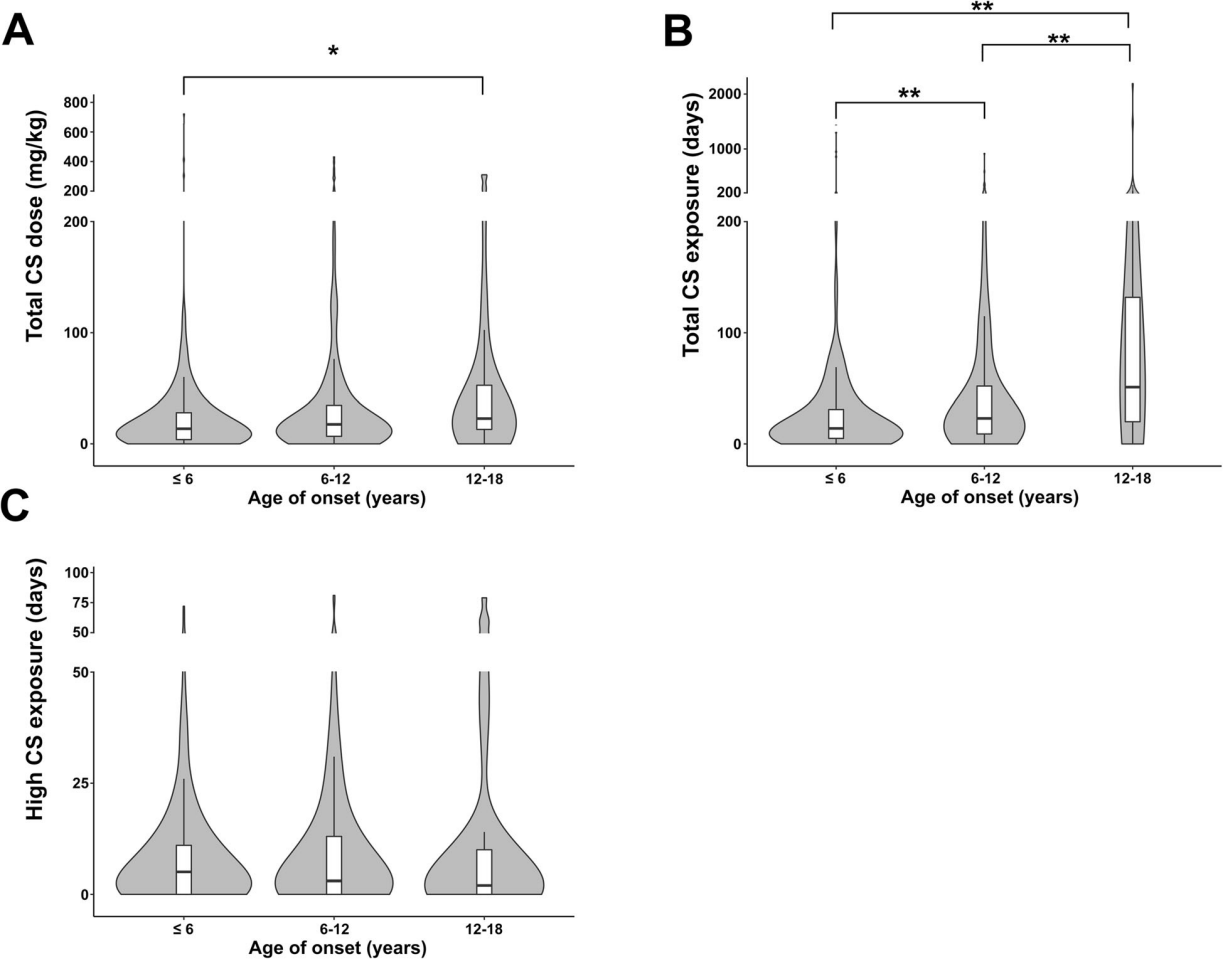
Distribution of total corticosteroid (CS) cumulative dose and exposure duration among different onset age groups. Violin plots showing distribution of **a** total CS cumulative dose, **b** total CS exposure duration, and **c** high dose CS exposure duration in different onset age groups: ≤ 6 years old, 6–12 years old (> 6, ≤ 12), and 12–18 years old (> 12, < 18). *P < 0.05, and **P < 0.001

Meanwhile, the cumulative CS dose (body weight adjusted equivalent dose of oral prednisolone) was significantly higher in the 12–18 years old group (median: 22.80 mg/kg, IQR: 13.05–52.65) compared with patients ≤6 years old (median: 13.83 mg/kg, IQR: 3.89–29.17) (Fig. 1a) (*P* = 0.011). The total CS exposure duration was also significantly longer in the 12–18 years old group (≤ 6 years old, median: 14 days, IQR: 5–32; 6–12 years old, median: 23 days, IQR: 9–52.8; 12–18 years old, median: 62 days, IQR: 25–156.5; *P* <  0.001), but the number of days under high dose exposure showed no difference (*P* = 0.681) (Fig. 1b and c).

To investigate clinical predictors of DMARD use, we regrouped the patients into three groups: no DMARDs required, DMARDs required, and DMARDs dependent. There were 253 (52.3%) patients who required DMARDs, and 76 of them were DMARDs dependent. Patients with older onset age, gastrointestinal involvement, and renal involvement had higher odds of requiring DMARDs; meanwhile, patients with older onset age and renal involvement were more prone to be DMARDs dependent (see Additional file 1: Table S2-S5).

Medication-associated major adverse effects occurred in some patients under azathioprine and cyclosporine. Neutropenia was observed in three cases (1.3%) and pancytopenia in two cases (0.9%) under azathioprine. Three (4.7%) patients with renal involvement developed hypertension under the combination of cyclosporine and CS. Apart from CS and/or cyclosporine, the disease process itself could have contributed to the development of hypertension, as underlying inflammatory renal disease has been associated with hypertension [18]. Gum swelling was documented in one (1.7%) patient taking cyclosporine.

### Recurrence, CS dependence, and refractory disease

The overall recurrence rate of IgAV was 9.5%, and recurrence was more frequent in patients older than 6 years old (≤ 6 years old, 7.3%; 6–12 years old, 9.5%; 12–18 years old, 22.5%; *P* = 0.0017). CS dependence was defined as CS exposure for more than 6 weeks in any IgAV episode. The CS dependence rate in 12–18 years old (55.0%) was significantly higher than the other two groups (≤6 years old, 19.2%; 6–12 years old, 32.9%; *P* < 0.001). The refractory rate showed similar patterns (≤ 6 years old, 7.3%; 6–12 years old, 17.6%; 12–18 years old, 55.0%; *P* < 0.001) (Table 5).

**Table 5:** Recurrence rate, corticosteroid dependence rate, and refractory disease rate in pediatric IgA vasculitis patients of different onset age

	Total (*n* = 484)	≤6 Y (*n* = 234)	6–12 Y (*n* = 210)	12–18 Y^a^ (*n* = 40)	*P* value
Recurrence^b^ rate	46 (9.5%)	17 (7.3%)	20 (9.5%)	9 (22.5%)	**0.010**
CS dependence^c^ rate	136 (28.1%)	45 (19.2%)	69 (32.9%)	22 (55.0%)	**< 0.001**
Refractory disease^d^ rate	76 (15.7%)	17 (7.3%)	37 (17.6%)	22 (55.0%)	**< 0.001**

Data shown are number (%) of patients

*Abbreviations*: *CS* Corticosteroid

^a^ Patients were grouped by onset age: ≤ 6 Y (years old), 6–12 Y (> 6, ≤ 12 years old), and 12–18 Y (> 12, < 18 years old)

^b^ Recurrence was defined as disease flare-up after complete remission and medication free for at least three months

^c^ CS dependence was defined as more than 6 weeks of daily oral CS intake

^d^ Refractory disease was defined as not achieving complete remission 6 months after disease onset

To further determine the major factors in recurrence, refractory disease and CS dependence, we performed univariate logistic regressions. Significant factors by univariate analysis were then entered into multivariate logistic regressions. By multivariate analysis, renal involvement was associated with a 3.05-fold (95% CI, 1.42–6.49; *P* = 0.004) increased odds of IgAV recurrence, a 20.51-fold (95% CI, 10.73–41.84; *P* < 0.001) increased odds of refractory disease, and a 5.46-fold (95% CI, 2.96–10.22; *P* < 0.001) increased odds of developing CS dependence compared to those without renal involvement (see Additional file 1: Table S6-S8).

Meanwhile, an increase of onset age by 1 year was associated with a 1.27-fold (95% CI, 1.16–1.39; P < 0.001) increased odds in developing refractory disease and a 1.12-fold (95% CI, 1.03–1.24; *P* = 0.012) increased odds of being CS dependent (see Additional file 1: Table S7, S8).

**Fig. 2:**
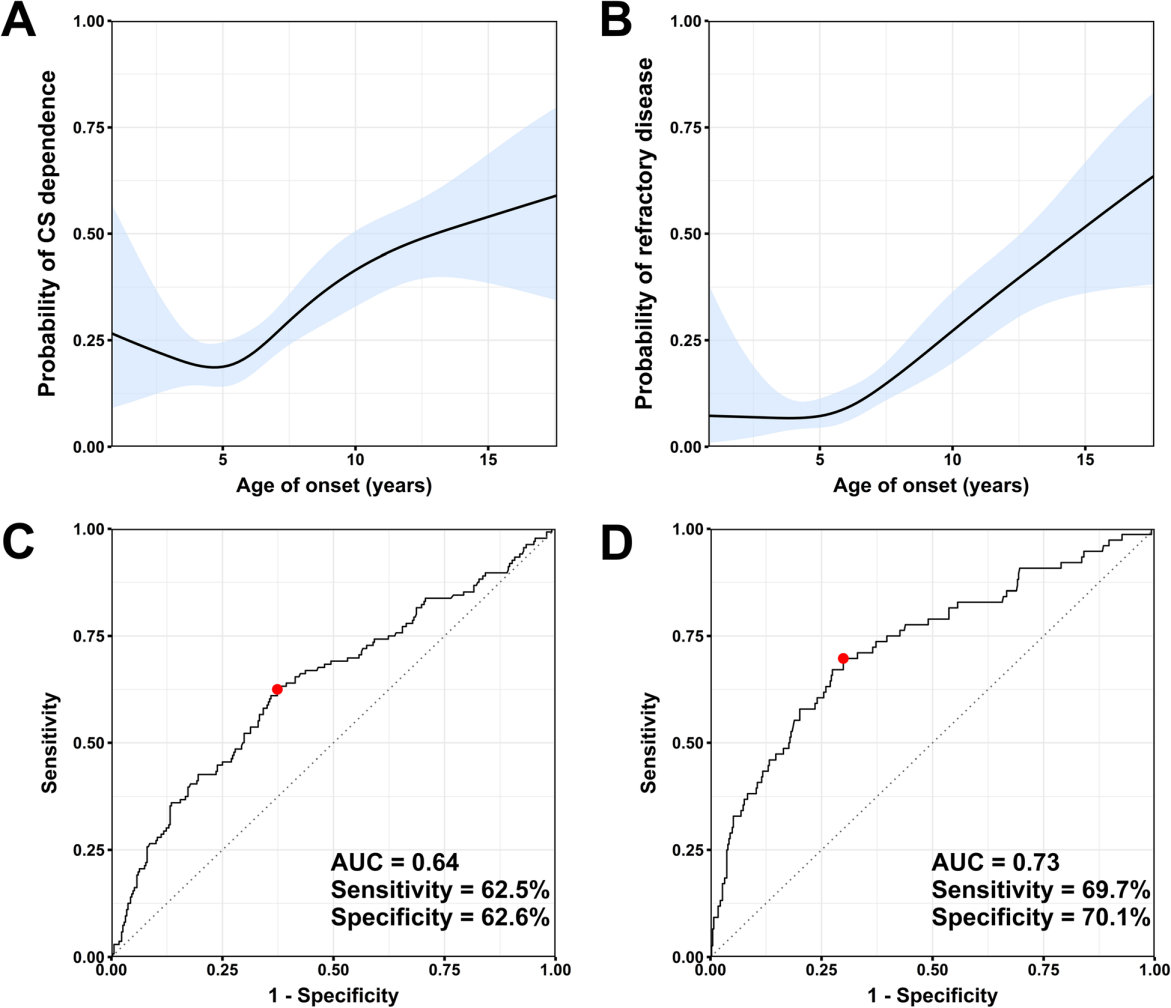
Association between onset age and corticosteroid dependence / refractory disease. Probability of **a** corticosteroid (CS) dependence or **b** refractory disease plotted against onset age, derived from a restricted cubic spline model. The shaded area represents the 95% confidence interval. ROC curve using onset age to distinguish patients with **c** CS dependent IgAV or **d** refractory IgAV compared with patients without CS dependence or refractory disease. The sensitivity and specificity are shown at the optimal diagnostic cutoff of onset age (red dot), which is **c** ≥ 6.5 years old for CS dependent IgAV and **d** ≥ 7.2 years old for refractory IgAV

To explore whether the risk of developing CS dependence and refractory disease increased linearly with onset age, we modeled the relationship between onset age and these outcomes with restricted cubic spline models. Figure 2 presents the fitted models for the probability of CS dependence and refractory disease at different onset ages. We found that the slope of the curve for CS dependence seemed to become steeper near 6.5 years old (Fig. 2a). At the optimal threshold established by ROC curve analysis (onset age ≥ 6.5 years old), the sensitivity of onset age to predict CS dependence was 62.5%, and the specificity was 62.6%, with an AUC of 0.64 (Fig. 2c).

The slope of the probability of refractory disease appeared to change near 7.5 years old (Fig. 2b). At the optimal threshold determined by ROC curve analysis (age ≥ 7.2 years old), the sensitivity of onset age to predict refractory disease was 69.7%, and the specificity was 70.1%, with an AUC of 0.73 (Fig. 2d).

## Discussion

To our knowledge, this is the largest study to demonstrate a distinct clinical course and outcome in children with IgAV of different onset ages. We showed that as onset age increased, patients had arthralgia or arthritis less frequently, but were more prone to renal involvement. Meanwhile, the risk of developing CS dependence increased with onset age, especially in patients older than 6.5 years old. A similar pattern was observed in risk of refractory disease, with a cut point of 7.2 years old. Patients with renal involvement had a higher risk of disease recurrence, but onset age did not impact the recurrence rate significantly.

In IgAV, severe GI involvement, such as massive GI bleeding and intussusception, might lead to mortality. In our series, 64.3% of patients had GI involvement, while 13.5% of them presented with GI bleeding. There was no significant difference of GI involvement rate in different age subgroups. In a previous study conducted in Taiwan from 1991 to 2001, the incidence of GI involvement and GI bleeding were 58 and 17.6%, respectively [19], which was similar to our data. The study also identified three cases with severe GI complications (one with intussusception, one with GI perforation, and one with massive GI bleeding induced hypovolemic shock); however, none of these complications were observed in our study. This might be attributed to increased awareness of IgAV complications and prompt treatment. Alternatively, it could echo the observation that clinical features of IgAV demonstrated a trend of progressively less severe phenotypes over the past three decades in Korea [20].

Renal involvement was noted in 25–56% of pediatric patients with IgAV [8, 12]. In our study, renal involvement developed in 26.9% of patients, and it occurred more frequently as onset age increased. Previous studies showed that pediatric patients over 10 years old stood a higher risk of renal involvement and severe renal disease [21, 22]. However, our analysis demonstrated that the risk of renal involvement appeared to increase continuously with onset age (see Additional file 1: Fig. S1).

As onset age increased, not only clinical manifestations but also laboratory data differed. To adjust for the confounding effect of different normative values for the hemogram and IgA at different ages, we subjected age-corrected Z-scores to further analysis. The mean Z-scores of WBC and IgA were positive in all three subgroups, indicating that the WBC count and IgA of patients with IgAV were higher than normal range irrespective of age, possibly reflecting the important roles of WBC and IgA in the pathogenesis in IgAV. As onset age increased, the Z-score of WBC increased, but the Z-score of IgA decreased. Though the mechanism remains unclear, we hypothesized that the relative importance of WBC and IgA in pathogenesis of IgAV changes in patients of different onset age. The importance of WBC might increase as onset age increases, and could possibly contribute to the higher frequency of renal involvement and refractory disease in elder children, but further studies are warranted.

The disease course of pediatric IgAV is usually benign, but 14–66% patients experience recurrence [12, 23–26]. The variation in the reported recurrence rate could be related to the discrepant definitions of recurrence used in different studies. In our analysis, the overall recurrence rate was 9.5%, which is lower compared to previous studies. It is possibly attributable to the relatively stringent definition of recurrence in our study design (reappearance of symptoms or signs 3 months after discontinuation of medications). For instance, a prospective study of 74 Greek children with IgAV reported the highest rate of recurrence (66%) with a more lenient definition (reappearance of symptoms of signs after a 2-week asymptomatic period) [26]. Factors impacting the recurrence of IgAV in previous studies included older age (age > 8 years old) and renal involvement [27]. In our study, only renal involvement was associated with a significantly higher risk of recurrence, while onset age showed only trend-level association.

With the self-limited nature of IgAV, most cases resolve within 4 weeks [28]. CS and/or DMARDs are usually considered in patients with severe visceral involvement, though there is a lack of randomized controlled trials demonstrating efficacy [29]. Treatment with CS might lessen the severity and duration of GI, joint, and cutaneous symptoms [28, 30], but does not alter the disease course nor prevent renal involvement [27, 31, 32]. In the literature, around 19.8–73.2% patients required CS use [12, 33, 34], while 0–75.9% patients took DMARDs [12, 34]. The variation in the reported administration rate of CS and DMARDs could relate to differences in disease severity and clinical characteristics in different studies. The preferred treatment protocol for pediatric IgA vasculitis might also vary between different medical institutions. Administration rate of CS and DMARDs in our study were 83.9 and 53.2% respectively. CS administration rate was especially high, compared to previous studies. This could possibly be attributed to practice differences or referral bias. In our institution, as a tertiary referral center, often cares for patients with higher disease severity or those transferred from local hospitals due to recalcitrant disease course. These patients often required more aggressive treatment.

Most physicians avoid extended CS administration, due to adverse effects of long-term CS use, such as osteoporosis, weight gain, and growth retardation. Some patients still require long-term CS use and/or additional DMARDs because of persistent active disease status under CS or disease relapse during CS tapering. In our study, the overall rates of CS dependence and refractory disease were 28.1 and 15.7%, respectively. Both increased with increasing onset age and were elevated in patents with renal involvement. Only a few previous studies addressed these issues. Alfredo et al. reported that 7.2% of IgAV patients had persistent active disease status for a period of 1 year or longer [25]. The total CS burden was highest in the 12–18 years old group, as measured by total CS dose or exposure days. To our knowledge, this has not been mentioned in previous literature.

In this study, the most commonly used DMARDs was azathioprine. Azathioprine, the most frequently prescribed, has been documented to achieve beneficial renal outcomes in alleviating histopathological change and shortening the clinical course of severe IgAV nephritis in combination with CS, though limited case numbers [35]. The major adverse effects of azathioprine include liver toxicity and bone marrow suppression. In our study, no patients developed liver enzyme abnormalities, but neutropenia and pancytopenia were observed. Regular monitoring of complete blood cell count and liver enzymes is indicated in patients under azathioprine.

Novel drugs with higher safety and efficacy in achieving CS sparing and preventing relapse are desired. Rituximab, a monoclonal antibody which depletes B cell by targeting CD20 [36], was reported to be beneficial for eight chronic steroid-dependent cases in reducing oral CS burden [37]. In our study, adjuvant rituximab treatment was administered in one case with persistent active disease status (arthritis and abdominal pain) despite CS and multiple DMARDs for 10 months. Partial remission was achieved after rituximab treatment.

There were several limitations in this cohort study. Given its retrospective nature, variable thoroughness of documentation might cause difficulties in distinguishing arthritis from focal angioedema around joints resulting from vasculitis. In younger children, an inability to express themselves could compound this problem. Further image studies, such as soft tissue sonography may help to differentiate these two conditions. A second limitation is the referral bias due to our institution’s status as a tertiary referral hospital. Patients with higher disease severity may be overrepresented in our cohort, and our single center experience may not reflect the universal condition. Further confirmation of our findings by multicenter studies or nationwide databases are warranted. Finally, the possibility of false positive results could be increased due to multiple hypothesis testing.

This is the largest cohort study demonstrating that pediatric IgAV with different onset ages are associated with distinct clinical manifestations and outcomes (including total CS burden). We found that the probability of developing CS dependence, refractory disease, and renal involvement increased with onset age. This indicates that the course of pediatric IgAV is not as benign as previously thought, especially in adolescents. Early introduction of DMARDs or biological agents in IgAV patients with either CS dependence or refractory course might help reducing CS burden and adverse effects of long-term CS use. However, larger and preferably randomized controlled studies are still needed for further clarification.

## Conclusion

Pediatric IgAV with different onset ages are associated with distinct clinical manifestations and outcomes. The probability of developing corticosteroid dependence, refractory disease, and renal involvement increased with onset age.

Early introduction of DMARDs or biological agents in IgAV patients with either CS dependence or refractory course might help reducing CS burden and adverse effects of long-term CS use.

## Supplementary Information

**Additional file 1.**

## Data Availability

The datasets used in the study are available from the corresponding author on reasonable request.

## Abbreviations

IgAV: Immunoglobulin A vasculitis
IgA: Immunoglobin A
IL-8: Interleukin-8
GI: Gastrointestinal
CS: Corticosteroids
NSAIDs: Non-steroidal anti-inflammatory drugs
DMARDs: Disease-modifying anti-rheumatic drugs
RBCs: Red blood cells
ROC: Receiver operating characteristic
Hb: Hemoglobin
Plt: Platelet
WBC: White blood cell
NLR: Neutrophil to lymphocyte ratio
PLR: Platelet to lymphocyte ratio
IQR: Interquartile range
AUC: Area under curve
