# Supplementary material for: Onset age is a risk factor for refractory pediatric IgA vasculitis: a retrospective cohort study
Source: Pediatr Rheumatol Online J. 2020 Nov 10;18:86. doi: 10.1186/s12969-020-00480-3 (PMC7654143; doi:10.1186/s12969-020-00480-3)

Table S1. Onset season in pediatric IgA vasculitis patients in different onset age groups.

|  | Total (n=484) | ≤6 Y (n=234) | 6-12 Y (n=210) | 12-18 Y^a^ (n=40) | *P* value |
| --- | --- | --- | --- | --- | --- |
| Spring | 113 (23.3%) | 52  (22.2%) | 53  (25.2%) | 8  (20.0%) | 0.670 |
| Summer | 88  (18.2%) | 41  (17.5%) | 38  (18.1%) | 9  (22.5%) |  |
| Autumn | 142 (29.3%) | 69  (29.5%) | 65  (31.0%) | 8  (20.0%) |  |
| Winter | 140 (28.9%) | 72  (30.8%) | 54  (25.7%) | 14  (35.0%) |  |

Data shown is number (%) of patients as appropriate.

^a^ Patients were grouped by onset age: ≤ 6 Y (years old), 6-12 Y (>6, ≤ 12 years old), and 12-18 Y (>12, < 18 years old).

Table S2. Demographics and clinical manifestations in pediatric IgA vasculitis stratified by DMARD use.

|  | Total  (n=484) | No DMARDs needed (n=231) | DMARDs needed  (n=253) | DMARDs  dependent  (n=76) | *P* value |
| --- | --- | --- | --- | --- | --- |
| Onset age, years old | 6.10 (4.72-8.58) | 5.43 (4.41-7.09) | 6.97 (5.11-9.63) | 8.89 (6.38-12.33) | **<0.001** |
| Male sex | 252(52.1%) | 123 (53.2%) | 129 (51.0%) | 35 (46.1%) | 0.550 |
| Preceding infection | 304(62.8%) | 157 (68.0%) | 147 (58.1%) | 34 (44.7%) | **<0.001** |
| Manifestation |  | | | | |
| Skin purpura | 484 (100%) | 231 (100%) | 253 (100%) | 76 (100%) | NA |
| Arthralgia/arthritis | 361(74.6%) | 188 (81.4%) | 173 (68.4%) | 47 (61.8%) | **<0.001** |
| CNS/PNS involvement | 5 (1.0%) | 0 | 5 (2.0%) | 4 (5.3%) | NA |
| GI involvement | 311(64.3%) | 126 (54.5%) | 185 (73.1%) | 48 (63.2%) | **<0.001** |
| Abdominal pain | 294(60.7%) | 115(49.8%) | 178(70.4%) | 47(61.8%) | **<0.001** |
| Vomiting | 100(20.7%) | 31 (13.4%) | 69 (27.3%) | 21 (27.6%) | **<0.001**. |
| Diarrhea | 34 (7.0%) | 12 (5.2%) | 21 (8.3%) | 6 (7.9%) | 0.3839 |
| GI bleeding | 42 (8.7%) | 6 (2.6%) | 36 (14.2%) | 12 (15.8%) | **<0.001** |
| Positive stool occult blood | 111(22.9%) | 33 (14.3%) | 78 (30.8%) | 21 (27.6%) | **<0.001** |
| Renal involvement | 130(26.9%) | 29 (12.6%) | 101 (39.9%) | 62 (81.6%) | **<0.001** |
| Microscopic hematuria | 129(26.7%) | 29 (12.6%) | 100 (43.3%) | 62 (81.6%) | **<0.001** |
| Gross hematuria | 10 (20.7%) | 0 | 10 (4.0%) | 10 (13.2%) | NA |
| Non-nephrotic proteinuria | 24 (5.0%) | 4 (1.7%) | 20 (7.9%) | 15 (19.7%) | **<0.001** |
| Nephrotic syndrome | 8 (1.7%) | 0 | 8 (3.2%) | 7 (9.2%) | NA |

Data shown are median (IQR) or number (%) of patients as appropriate.

Abbreviations: IgA, immunoglobulin A; DMARD, disease-modifying anti-rheumatic drugs; CNS, central nervous system; PNS, peripheral nervous system; GI, gastrointestinal; NA, not available.

Table S3. Adjusted laboratory parameters in pediatric IgA vasculitis patients stratified by DMARD use.

|  | No DMARDs required  (n=231) | DMARDs  required  (n=253) | DMARDs  dependent  (n=76) | *P* value |
| --- | --- | --- | --- | --- |
| Hb Z score | -0.34±1.78  (n=188) | -0.03±1.64  (n=175) | -0.27±1.29  (n=46) | 0.154 |
| WBC Z score | 1.98±1.85  (n=189) | 2.43±2.18  (n=173) | 2.33±2.23  (n=45) | 0.124 |
| Neutrophil Z score | 1.77±2.10  (n=185) | 2.26±2.55  (n=167) | 1.97±2.28  (n=40) | 0.286 |
| Lymphocyte Z score | -0.01±1.29  (n=184) | -0.08±1.45  (n=167) | -0.01±1.17  (n=40) | 0.639 |
| NLR | 2.05 (1.36-3.14)  (n=184) | 2.19 (1.57-4.52)  (n=167) | 2.13 (1.60-4.74)  (n=40) | **0.020** |
| PLR | 119.38 (87.50-152.95)  (n=183) | 124.15 (89.41-181.11)  (n=166) | 130.55 (102.20-166.27)  (n=39) | 0.163 |
| IgA Z score | 4.11±3.28  (n=159) | 3.28±2.72  (n=193) | 3.13±3.54  (n=53) | **0.007** |

Data are presented as median (IQR) or mean ± SD as appropriate

Abbreviations: DMARD, disease-modifying anti-rheumatic drugs; Hb, hemoglobin; Plt, platelet; WBC, white blood cells; PLR, platelet-to-lymphocyte ratio; NLR, neutrophil-to-lymphocyte ratio; IgA, immunoglobulin A

Table S4. Factors associated with DMARD use in in pediatric IgA vasculitis patients.

|  | Univariate | | | Multivariate | | |
| --- | --- | --- | --- | --- | --- | --- |
|  | OR | 95% CI | *P* value | OR | 95% CI | *P* value |
| Onset age | 1.18 | 1.11–1.26 | **<0.001** | 1.17 | 1.07–1.29 | **0.001** |
| Preceding infection | 0.65 | 0.45-0.95 | **0.025** | 1.01 | 0.57-1.79 | 0.978 |
| Arthralgia/arthritis | 0.49 | 0.32–0.75 | **0.001** | 0.99 | 0.51-1.93 | 0.988 |
| GI involvement | 2.27 | 1.55-3.33 | **<0.001** | 2.45 | 1.37-4.44 | **0.003** |
| Renal involvement | 4.63 | 2.95–7.46 | **<0.001** | 4.06 | 2.14-8.00 | **<0.001** |
| NLR | 1.13 | 1.04–1.24 | **0.006** | 1.00 | 0.90-1.11 | 0.958 |
| IgA Z score | 0.91 | 0.84–0.98 | **0.011** | 0.94 | 0.85-1.03 | 0.208 |

Abbreviations: DMARD, disease-modifying anti-rheumatic drugs; OR, odds ratio; CI, confidence interval; GI, gastrointestinal; NLR, neutrophil-to-lymphocyte ratio; IgA, immunoglobulin A

Table S5. Factors associated with DMARDs dependent in pediatric IgA vasculitis patients.

|  | Univariate | | | Multivariate | | |
| --- | --- | --- | --- | --- | --- | --- |
|  | OR | 95% CI | *P* value | OR | 95% CI | *P* value |
| Onset age | 1.29 | 1.20–1.39 | **<0.001** | 1.26 | 1.15-1.39 | **<0.001** |
| Preceding infection | 0.41 | 0.25-0.68 | **<0.001** | 0.71 | 0.38-1.34 | 0.288 |
| Arthralgia/arthritis | 0.49 | 0.29–0.82 | **0.006** | 1.24 | 0.63-2.51 | 0.534 |
| GI involvement | 0.95 | 0.57-1.59 | 0.828 |  |  | - |
| Renal involvement | 22.14 | 12.05–43.30 | **<0.001** | 19.73 | 10.30-40.28 | **<0.001** |
| NLR | 1.03 | 0.92–1.12 | 0.553 |  | - | - |
| IgA Z score | 0.92 | 0.82–1.03 | 0.169 |  | - | - |

Abbreviations: DMARD, disease-modifying anti-rheumatic drugs; OR, odds ratio; CI, confidence interval; GI, gastrointestinal; NLR, neutrophil-to-lymphocyte ratio; IgA, immunoglobulin A

Table S6. Factors associated with recurrent IgA vasculitis.

|  | Univariate | | | Multivariate | | |
| --- | --- | --- | --- | --- | --- | --- |
|  | OR | 95% CI | *P* value | OR | 95% CI | *P* value |
| Onset age | 1.14 | 1.04–1.23 | **0.003** | 1.11 | 1.00–1.23 | 0.050 |
| Arthralgia/arthritis | 0.85 | 0.44–1.73 | 0.641 | - | - | - |
| Renal involvement | 2.80 | 1.50–5.20 | **0.001** | 3.05 | 1.42–6.49 | **0.004** |
| Hb Z score | 1.13 | 0.93–1.37 | 0.224 | - | - | - |
| WBC Z score | 1.15 | 0.99–1.32 | 0.064 | - | - | - |
| NLR | 1.09 | 1.00–1.19 | **0.040** | 1.04 | 0.95–1.14 | 0.354 |
| PLR | 1.00 | 1.00–1.01 | 0.223 | - | - | - |
| IgA Z score | 0.91 | 0.79–1.03 | 0.177 | - | - | - |

Abbreviations: OR, odds ratio; CI, confidence interval; Hb, hemoglobin; WBC, white blood cells; PLR, platelet-to-lymphocyte ratio; NLR, neutrophil-to-lymphocyte ratio; IgA, immunoglobulin A.

Table S7. Factors associated with refractory IgA vasculitis.

|  | Univariate | | | Multivariate | | |
| --- | --- | --- | --- | --- | --- | --- |
|  | OR | 95% CI | *P* value | OR | 95% CI | *P* value |
| Onset age | 1.29 | 1.20-1.39 | **<0.001** | 1.27 | 1.16-1.39 | **<0.001** |
| Arthralgia/arthritis | 0.49 | 0.29-0.82 | **0.006** | 1.22 | 0.62-2.45 | 0.571 |
| Renal involvement | 22.14 | 12.05-43.30 | **<0.001** | 20.51 | 10.73-41.84 | **<0.001** |
| Hb Z score | 0.97 | 0.81-1.16 | 0.731 | - | - | - |
| WBC Z score | 1.04 | 0.89-1.19 | 0.625 | - | - | - |
| NLR | 1.03 | 0.93-1.12 | 0.553 | - | - | - |
| PLR | 1.00 | 1.00-1.00 | 0.417 | - | - | - |
| IgA Z score | 0.92 | 0.82-1.03 | 0.169 | - | - | - |

Abbreviations: OR, odds ratio; CI, confidence interval; Hb, hemoglobin; WBC, white blood cells; PLR, platelet-to-lymphocyte ratio; NLR, neutrophil-to-lymphocyte ratio; IgA, immunoglobulin A.

Table S8. Factors associated with CS dependent IgA vasculitis.

|  | Univariate | | | Multivariate | | |
| --- | --- | --- | --- | --- | --- | --- |
|  | OR | 95% CI | *P* value | OR | 95% CI | *P* value |
| Onset age | 1.17 | 1.10-1.24 | **<0.001** | 1.12 | 1.03-1.24 | **0.012** |
| Arthralgia/arthritis | 0.68 | 0.44-1.06 | 0.085 | - | - | - |
| Renal involvement | 5.35 | 3.47-8.33 | **<0.001** | 5.46 | 2.96-10.22 | **<0.001** |
| Hb Z score | 1.07 | 0.93-1.24 | 0.338 | - | - | - |
| WBC Z score | 1.13 | 1.00-1.27 | **0.039** | 1.03 | 0.89-1.19 | 0.715 |
| NLR | 1.07 | 1.00-1.16 | 0.057 | - | - | - |
| PLR | 1.00 | 1.00-1.00 | 0.580 | - | - | - |
| IgA Z score | 0.90 | 0.82-0.98 | **0.026** | 0.89 | 0.77-1.01 | 0.084 |

Abbreviations: CS, corticosteroid; OR, odds ratio; CI, confidence interval; Hb, hemoglobin; WBC, white blood cells; PLR, platelet-to-lymphocyte ratio; NLR, neutrophil-to-lymphocyte ratio; IgA, immunoglobulin A

**FIGURE LEGEND**

Fig S1. Results of a restricted cubic spline model showing the association between onset age and the probability of developing renal involvement.

**Figure S1**


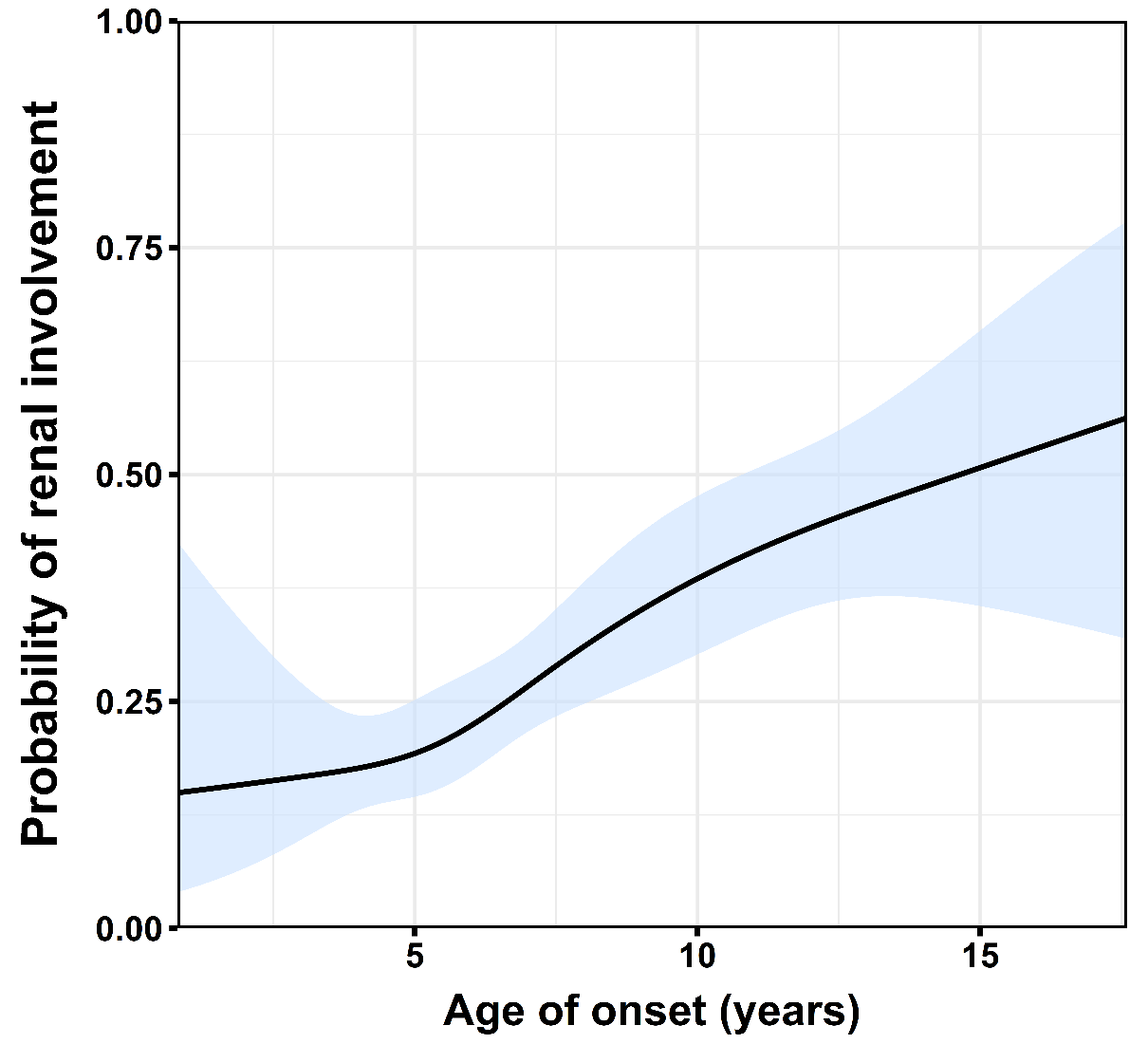

Supplement: Supplementary file 1 — Additional file 1. [file 12969_2020_480_MOESM1_ESM.docx]
